# Career advising in family medicine: a theoretical framework for structuring the medical student/faculty advisor interview

**DOI:** 10.3402/meo.v18i0.21173

**Published:** 2013-08-13

**Authors:** Melissa Bradner, Steven H. Crossman, Allison A. Vanderbilt, Judy Gary, Paul Munson

**Affiliations:** 1 Department of Family Medicine, Virginia Commonwealth University School of Medicine, Richmond, VA, USA; 2 Assessment and Evaluation Center on Health Disparities, Virginia Commonwealth University School of Medicine, Richmond, VA, USA

**Keywords:** career choice specialties, medical decision-making education, medical education, undergraduate medical education

## Abstract

**Background:**

There are unique challenges to recruiting students into the specialty of family medicine within academic medical centers.

**Methods:**

At Virginia Commonwealth University, we developed an advising framework to help students address institutional and personal obstacles to choosing family medicine as a career.

**Results:**

The role of a faculty advisor is not to direct the student to a career choice but rather to foster a mentor relationship and help the student come to his or her own realizations regarding career choice. The faculty advisor/medical student interview is conceptualized as five discussion topics: self-knowledge, perception, organizational voice, cognitive dissonance, and anticipatory counseling.

**Conclusion:**

This framework is intended to assist faculty in their efforts to encourage students to consider a career in family medicine.

There are unique challenges to recruiting students into the specialty of family medicine in academic medical centers. This article presents a framework to structure the medical student/faculty advisor encounter that takes into consideration the paradox of recruiting medical students for a predominately community-minded specialty in the setting of a large academic medical center (1). Studies suggest that the specialty of family medicine does not have the image and credibility it merits within academic medical centers (2) to foster and produce future family medicine physicians. Though there has been a slight increase in enrollment in family medicine residency in the last few years, the added demands for family physicians as a result of the implementation of the Patient Protection and Affordable Care Act (ACA) and the impact of those demands on specialty choice remain to be seen. There is a strong likelihood that the United States will face a primary care physician shortage (3). Recent efforts at predicting this need estimate that roughly 8,000 primary care physicians are needed as a result of the insurance expansion and an even greater number of primary care physicians (33,000) are needed by 2025 to care for the US growing and aging population (4). Factors affecting student selection of family medicine as a career include: admissions committees selecting students who are more likely to choose other specialties, students having greater exposure to specialist teachers and role models, limited exposure to primary care clinical experience and medical specialty ‘bashing’ (5). Bashing is defined as negative feedback and comments towards a particular medical specialty (6).

Criticism of medical specialties occurs frequently within medical teaching facilities and is not exclusive to the specialty of family medicine. In a recent survey of students applying to dermatology residency, 48% experienced belittlement of their specialty choice (7). The impact on family medicine is more pernicious given the public health implications of inadequate primary care workforce recruitment. Bashing of family medicine can come from a student's family, friends, medical school peers, residents, attending physicians, and even family physicians themselves (8). The impact of bashing on career choice has not been sufficiently recognized and addressed by medical school faculty. A study done by Holmes and colleagues that surveyed 105 medical students showed that students reported bashing in all six clerkships. The majority of bashing occurred during the surgery rotation (87.5%), with family medicine being the primary target most often (72%) (6). As role models, physicians can significantly influence students’ decisions about what specialty to pursue. Negative criticism towards family medicine can easily deter a student away from this discipline. One study reported that 84% of medical students changed their career choice due to negative comments about their specialty of interest (9).

## Faculty advisor implementation

At Virginia Commonwealth University, we developed an advising framework to help students address institutional and personal challenges to choosing family medicine as a career. The role of a faculty advisor is not to direct the student to a career choice but to foster a mentoring relationship and help the student come to his or her own realizations about career choice. The faculty advisor guides the student through two processes: (1) information gathering and giving on behalf of the medical student and (2) decision making. This allows the student to speak freely with a faculty advisor to decide what medical specialty is the best choice for the individual medical student.

Information gathering and giving takes place on an ongoing basis and is composed of a collection of facts, beliefs and opinions that might influence a medical student's decision. Both the faculty advisor and the student jointly participate in this process of sharing and gathering of information. The medical student independently makes the final decision about career choice. Some students may require additional time to talk through various specialties in medicine with their faculty advisor prior to making their final decision.

## Framework

**Fig. 1:**
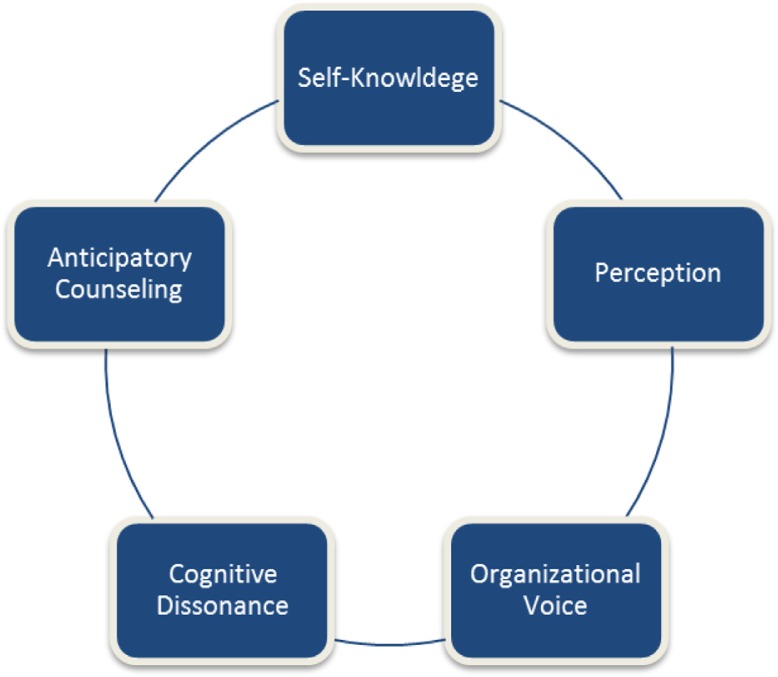
Diagramatic flow of 5 career advising discussion topics.

This framework proposes that any student statements or questions need to be viewed as a starting point – a bridge – to exploring in further depth the student's knowledge and interests. The key role for the family medicine faculty advisor is to ask the right questions. This process of inquiry is organized into five conceptual areas: (1) self-knowledge; (2) perception; (3) organizational voice; (4) cognitive dissonance; and (5) anticipatory counseling. Refer to Fig. 1 for the theoretical framework.

### Self-knowledge

The self-knowledge approach to advising seeks to lead students to their own conclusions by exploring the students’ personal interests, abilities and career values. Self-knowledge is the understanding of one's own nature, preferences, priorities, abilities and limitations. In a literature review conducted by Reed and colleagues (10), it was reported that ‘students generally made specialty choice decisions on the basis of images of themselves and of medicine as a whole rather than on specific knowledge of particular specialties.’

Students interested in family medicine reported that they are willing to earn less income in order to gain the lifestyle of flexibility, scope of practice, and enduring patient relationships they desired (8). Students rejecting family medicine were more likely to cite insufficient prestige, low intellectual content, and concern about mastering a broad content area as reasons for rejection (11).

Self-knowledge questions are designed to open a discussion of the student's desire for future achievements, life style, status, salary and commitment to service, as well as to explore the student's sense of purpose and meaning of work. The self-knowledge key inquiry questions are: (1) what makes you want to go to work every day? (2) what energizes you? (3) what drains you? and (4) what really matters to you? More specifically, self-knowledge inquiry would be appropriate to address the following types of student statements or questions:

**Table:** 

Medical student statement	Faculty advisor inquiry
‘I think I like family medicine but I also did really well in OB.’	‘What is it that you enjoy about both of these specialties?’ ‘What energizes you, what drains you?’
‘I like working with my hands and think I would be good at surgery but I miss the patient interaction.’	‘What do you value most about your work?’

Students are encouraged to reflect on their thoughts and self-conceptions.

### Perception

The faculty advisor's role is to help students examine the perceptions they encounter from others about family medicine, and how these impact the student's own mental image of what family medicine is. Sometimes advisors need to provide information, correct misinformation or better yet, encourage students to consider perceptions (their own or other's) within a personal context and institutional context. The perception framework questions are designed to stimulate reflection about the information a student might hear that attempts to define or describe family medicine. Individuals who make comments about family medicine have various levels of understanding about what a career in family medicine entails. Messages may come from peers, family, society and family physicians themselves. While these influences may be positive or negative, the overall impact at present indicates that these issues related to perceptions contribute to an inadequate number of students choosing family medicine as a career choice (6).

Rather than telling the student the faculty advisor's perception, the inquiry framework asks the student to explore why the statement was made and how the student could get to the facts behind the statement. The perception key inquiry questions are: (1) on what information do you think he or she based that statement? and (2) what information would you need to make your own judgments? The perception inquiry would be appropriate for the following student statements or questions:

**Table:** 

Medical student statement	Faculty advisor inquiry
‘I heard family physicians get sued more often.’	‘How would you find out if that is indeed true?’
‘Faculty members say you don't make enough money in family medicine to pay back your loans.’	‘Whom could you speak with who might provide you with facts?’ ‘What do you think they mean by “enough”?’
‘Don't you only see cough and colds in family medicine?’	‘Why might someone have this perception?’
‘My microbiology professor told me I was too smart to go into family medicine.’	‘Why might the microbiology professor have that perception?’ ‘Where do they work?’ ‘What is their knowledge of family medicine?’ ‘What basis would they have to make this judgment?’

### Organizational voice

The concept of organizational voice is important to address with students in the context of academic medical centers and the social peer influence of a medical school class. There has been substantial discussion in the literature of the ‘hidden curriculum’ or unwritten behavioral code that underscores medical training, though is not defined in any syllabus or school learning objective but has a significant impact on student learning (12). One of those hidden assumptions is that specialty care is better than generalist care (13). Among students who enter medicine with an initial interest in family medicine, the hidden curriculum becomes most noticeable during the second year, when the majority of classes are taught by physician specialists (14). Pestiaux and Vanwelde expressed their opinions about the hidden curriculum in the Canadian Family Physician stating:

The condescending remarks and opinions of specialist teachers have led all their listeners into total ignorance of the existence, content, and specialized nature of general medicine which has become a fallback choice with no special attributes or particular use. (15)

Discussion of organizational oice in an advising setting attempts to illuminate for the student how group perspective, especially a small vocal group or one politically connected individual, can influence and color perception. One organizational voice key inquiry question is: how widely is this view held and by whom? The organizational voice inquiry would be appropriate for the following student statements or questions:

**Table:** 

Medical student statement	Faculty advisor inquiry
‘Everyone says that only the students at the bottom percentile of the class go into Family Medicine.’	‘How widely is this viewpoint held?’ ‘How have they come to hold this opinion?’ ‘What information would you need to form your own judgment?’

Unlike perception, which looks at the personal motivation of an individual to make a statement or propose an idea as fact, organizational voice addresses the culture of an institution and asks the student to reflect on the realities that might influence a widely held belief or a belief held by a vocal core group of formal or informal institutional leaders.

### Cognitive dissonance

Early on in their medical training, students with an interest in family medicine experience a state of cognitive dissonance: they believe that family medicine is a good fulfilling career choice but are confronted with beliefs that suggest otherwise (16). In subtle and not so subtle ways, messages are conveyed to students about a career in family medicine – ‘Why? You're so smart!’ ‘How will you pay back your loans?’ ‘Isn't that going to be taken over by nurse practitioners?’ When students experience negative comments about family medicine, they are left with a decision to either believe the statements or dismiss them as untrue. They are in a state of dissonance. But how can they dismiss the statements when they come from their professor?

As faculty advisors, we need to raise students’ awareness of this dissonance and address their concerns and anxiety. Advisors and family medicine departments need to become a source of information about the specialty of family medicine that is realistic and accurate. The advisor and family medicine department need to establish trust with interested students and follow them throughout their education. When the student encounters a negative comment from a professor, the student needs to reconcile this discrepancy with what they have experienced and understand about family medicine.

**Fig. 2:**
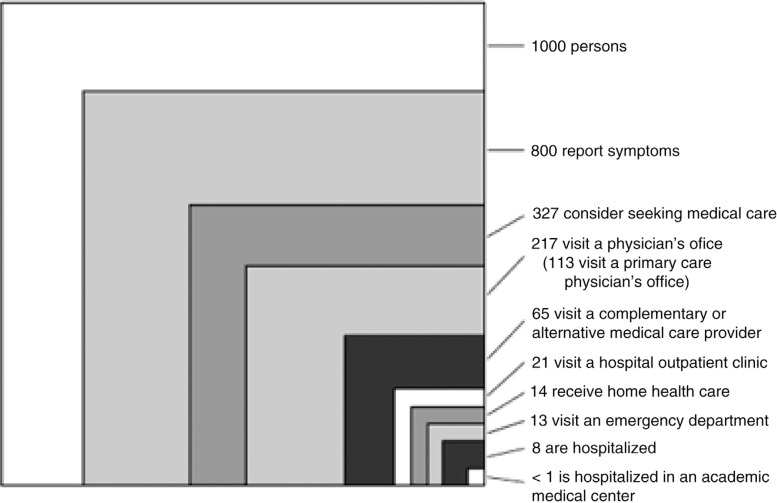
The ecology of medical care.

One way to frame the cognitive dissonance of the family medicine experience within academic specialty centers is to use the Kerr White graph from 1961, which was updated in a 2001 study. Both studies looked at the typical distribution of 1,000 adult patients over a 1-month period and where these adults received medical care. Remarkably, there was little change from 1961 to 2001: less than one patient out of 1,000 ends up being treated at an academic medical center. This graph may be used to illustrate the differences of the family medicine care environment in the community and the reality of the academic medical center (17). The profound differences between a community care environment and a medical specialty center can explain why an academic specialist might form a different judgment of family medicine than a specialist working in the community. Figure 2 shows that students spend the majority of their medical education in the academic center, which does not reflect the type of patients they will see in a community setting: students are learning medicine in the small box in the corner (<1), when in reality most medical care occurs in an office (box to the left).

Cognitive dissonance key inquiry questions: (1) in what ways is any community setting different from academic center culture? and (2) in what ways do you think practicing in a community setting might be different than an academic medical center? The cognitive dissonance inquiry would be appropriate for the following student statements or questions:

**Table:** 

Medical student statement	Faculty advisor inquiry
‘I hear that family physicians make more errors and do not provide good patient care.’	‘Why might that be the perception at an academic medical center?’ ‘Where could you find a reasonable source of information to find the answer to that question?’
‘No one respects family doctors.’	‘What experiences led you to this conclusion?’ ‘How would you verify if this statement is true or not?’ ‘Where and with whom would you want to confirm impressions of family doctors?’
‘I want to do something more challenging than general medicine.’	‘How did you form the perception that general medicine is not challenging?’ ‘How is practicing medicine in an academic hospital different than community practice?’

Cognitive dissonance is related to perception and organizational voice but in this advising scenario, the goal is to specifically examine the student's sense of dissonance of choosing a career that is not esteemed by peers and/or the student's training institution.

### Anticipatory counseling

Within every medical school class, there are informal and formal leaders who play a pivotal role in their social groups (18). Their beliefs are looked upon with high regard and their influence impacts students’ opinions: ‘In an environment like this, negative attitudes and misinformation are self perpetuating without strong, consistent messages to contest them (19).’ When medical students are exposed to negative impressions from their peers about family medicine, it may reinforce the pressure of going into a specialty practice, rather than primary care (8).

Anticipatory counseling seeks to foster students’ awareness that they might hear negative statements about family medicine and to prepare students for how they might react to these statements. The advisor needs to be conscious of the student's need for support and encouragement early in the advising relationship. Anticipatory counseling is a way to open a dialog around these personal and institutional barriers to choosing family medicine as a career. Anticipatory counseling key inquiry questions include: (1) how might you respond to faculty who actively criticize your choice (or consideration) of family medicine? and (2) what kinds of attitudes about family medicine might you expect to hear from other students, faculty, family, or friends?

## Discussion

The five conceptual areas of advising inquiry discussed (self-knowledge, perception, organizational voice, cognitive dissonance, and anticipatory counseling) are intended to be used in a fluid manner. A student statement might call for more probing about self-knowledge but then lead into a discussion of perception and organizational voice. A statement that leads to discussion of the differences between academic and community medical practice might lead to exploring with the student his or her thoughts on personal strengths and preferences.

Specialty ‘bashing’ is an unprofessional behavior not limited to family medicine. Medical schools need to address bashing in their efforts to improve professionalism training (12). On an institutional level, family medicine departments need to advocate for more resources that allow students to discover the day-to-day working reality of the specialty. For example, efforts to change student perception could include having students spend more time in community practice, as well as having more family physicians teach courses in medical school (19). Family medicine faculty should also be promoted to students in a positive light wherever possible to serve as ‘counterexamples to negative stereotypes (20).’

The process of student specialty selection is one of the most important and challenging aspects facing medical education today (9). Family medicine core values such as continuity of care and person-focused, holistic care are not typically emphasized within academic medical centers. The framework we have described is a way to grapple with these institutional realities and work with students on the conflicting messages they are likely to receive about family medicine over their 4 years of undergraduate medical education. We hope that this advising and inquiry process will assist family medicine faculty in their role as academic advisors and help them address the unique challenges our students face when considering a career in family medicine. The key role for the family medicine advisor is to ask the right questions and guide the career choice discussion.

## Appendix: Questions to Help Frame the Advisor/Advisee Career Discussion

The ***self-knowledge*** key inquiry questions are: (1) What makes you want to go to work every day? (2) What energizes you? (3) What drains you? and (4) What really matters to you?

**Table:**

Medical student statement	Faculty advisor inquiry
‘I think I like family medicine but I also did really well in OB?’	‘What is it that you enjoy about both of these specialties?’ ‘What energizes you, what drains you?

The *perception* key inquiry questions are: (1)on what information do you think he or she based that statement? and (2) what information would you need to make your own judgments?

**Table:**

Medical student statement	Faculty advisor inquiry
‘Faculty members say you don't make enough money in family medicine to pay back your loans.’	‘Whom could you speak with who might provide you with facts?’ ‘What do you think they mean by “enough”?’
‘My microbiology professor told me I was too smart to go into family medicine.’	‘Why might the microbiology professor have that perception, where do they work, what is their knowledge of family medicine?’

The *organizational voice* key inquiry question: how widely is this view held and by whom?

**Table:**

Medical student statement	Faculty advisor inquiry
‘Everyone says that only the students at the bottom percentile of the class go into Family Medicine.’	‘How widely is this viewpoint held?’ ‘How have they come to hold this opinion?’ ‘What information would you need to form your own judgment?’

*Cognitive dissonance* key inquiry questions: (1) in what ways is any community setting quality different from academic center culture? (2) in what way do you think practicing in a community setting might be different than an academic medical center?

**Table:**

Medical student statement	Faculty advisor inquiry
‘No one respects family doctors.’	‘What experiences led you to this conclusion?’ ‘How would you verify if this statement is true or not?’ ‘Where and with whom would you want to confirm impressions of family doctors?’

*Anticipatory advising* key inquiry question (1) how might you respond to faculty who actively criticize your choice (or consideration) of family medicine? and (2) what kinds of attitudes about family medicine might you expect to hear from other students, faculty, family, or friends?
